# Idiopathic pulmonary fibrosis: moving forward

**DOI:** 10.1186/s12916-015-0481-6

**Published:** 2015-09-24

**Authors:** Luca Richeldi

**Affiliations:** University of Southampton, Southampton, UK; Southampton Respiratory Biomedical Research Unit, Mailpoint 813, LE75 E Level, South Academic Block, Southampton, UK; University Hospital Southampton NHS Foundation Trust, Southampton, SO16 6YD UK

**Keywords:** Idiopathic pulmonary fibrosis, Interstitial lung disease, High-resolution computed tomography, Patient-reported outcomes, Epidemiology, Staging

## Abstract

Idiopathic pulmonary fibrosis (IPF) is the prototype of a large and heterogeneous group of pulmonary disorders, mainly chronic and progressive, usually known as interstitial lung disease (ILD). Over the last few decades, IPF has been increasingly recognized as a major unmet medical need in respiratory medicine and has become the focus of intense research activity. This is due to the fact that IPF incidence is increasing worldwide, with rates (and unfortunately prognosis) which are very similar to those of many forms of cancer. Basic and clinical research on IPF has been enormously advancing over the last few decades, culminating in the recent discovery of two safe and effective drugs, now finally made available to patients. For all these reasons, missing a diagnosis of IPF is not acceptable anymore and there is a need for spreading the knowledge about IPF across various specialties of medicine globally. In this context, this article collection in *BMC Medicine* contributes to the ultimate goal of early identification and better management of patients with ILD, and IPF in particular, worldwide.

## Introduction

The landscape of respiratory medicine has been transformed over the last few decades by the emergence of a group of variegated diseases, commonly referred to as interstitial lung diseases (ILDs). Once considered rare, difficult to diagnose and substantially untreatable, today ILD still represents a medical challenge. We know now that IPF incidence is increasing worldwide and rates are coming together across countries. Recent evidence suggests that incidence of IPF is increasing worldwide and it is very similar to that of conditions such as liver cancer [1]. However, mainly thanks to the advancements in the field of chest imaging and to the initial successes in the search for safe and effective drugs, ILDs are progressively becoming part of the daily clinical practice for specialists in respiratory medicine, geriatrics, internal medicine, thoracic radiology, pathology and surgery. Idiopathic pulmonary fibrosis (IPF) is the prototype of ILD, given its prevalence and poor prognosis. Current management of IPF patients is based on international evidence-based guidelines [2] and the accuracy of the diagnostic process is becoming crucially important since drugs have been approved worldwide [3, 4]. In this context and with a timely decision, the editorial team of *BMC Medicine*, the flagship medical journal of the *BMC* series, has invited a number of IPF experts worldwide to contribute commissioned pieces and original research articles for this special collection titled, *Idiopathic pulmonary fibrosis: diagnosis, management and new therapies*. Some particularly hot topics have been covered in this collection, which is being launched concomitantly with the opening of the 2015 International Congress of the European Respiratory Society. Other articles will also be featured in the collection and will be published in the next few weeks.

Few fields of modern respiratory medicine have been studied in their genetic component as IPF. In their Opinion article, Susan Mathai and her colleagues address the increasingly relevant topic of incorporating genetics in the diagnosis and the treatment of patients with IPF [5]. In addition to the existing genetic factors already known to contribute to disease risk and prognosis, more genetic variants will contribute to identify more precisely different phenotypes of the disease, characterized by different clinical behaviours and possibly by different responses to existing and future drugs. Genetic variants have not been included so far in the design of clinical trials; however, it is likely that the design of future studies will stratify patients based on their genetic background, or will at least pre-define subgroup analyses based on different genotypes. It will be of great interest to see how, in the next few years, genetic testing might help in identifying patients with early disease, particularly in the context of familial forms of the disease.

In addition to genetic factors, a number of other, largely unknown, determinants are causing IPF. As this disease is idiopathic by definition, answering the patient’s question of ‘why did I get IPF?’ is a major challenge for any physician. Reading the Commentary by Pierre-Simon Bellaye and Martin Kolb will help readers understand the complexity of the field, but also the recent and promising advancements achieved by the expanding community of basic and translational IPF researchers [6]. We have been moving far away from the concept of IPF being an inflammatory or immune-mediated disease, towards the new idea of an initially protective process leading to an uncontrolled, irreversible disruption of lung architecture and function. Fibrogenesis is now viewed as the key process (and therefore a potential therapeutic target) in IPF, although requiring the combined interaction of multiple factors which cause both epithelial injury and impaired wound healing. As such, IPF appears now as a prototype of progressive fibrosis, which is a leading cause of morbidity and mortality worldwide, and a potential bridge towards a therapeutic revolution in medicine.

Some patients, when diagnosed with IPF, have a sense of relief because they have not been diagnosed with lung cancer. Unfortunately, this sensation of good luck disappears when they discover that the two diseases, in their eyes so different, have a very similar prognosis. Carlo Vancheri in his Opinion article discusses the many (often hidden) links between IPF and cancer, thus highlighting the potential positive pitfalls of considering these two entities as closely inter-related [7]. In reality, most forms of cancer could be seen as ‘idiopathic’, and by comparing and contrasting IPF and cancer we might be able to identify more effectively shared mechanisms, with obvious therapeutic implications.

If it is a challenge discussing with IPF patients the causes of their disease, it is maybe even more difficult to address their expectations and anxieties with regard to their future. Kerri Johannson and colleagues have been exploring this topic over the last few years and they summarize the current status of knowledge in their Commentary [8]. Although it is still impossible to precisely predict the clinical trajectory for an individual IPF patient, sophisticated modelling of large retrospective cohorts allowed the identification of measurable factors, like gender, age and lung function levels, which provide an estimate of the future risk of mortality. Staging of IPF will help design more efficient clinical trials and identify groups of patients with more aggressive behaviour; this will also be important for counselling and early referral for lung transplant. The incorporation of genetic and molecular markers will undoubtedly help staging IPF patients, hopefully including predictors of response to treatments. In order to achieve this ambitious goal, centralized registries with systematically banked bio-samples will likely become instrumental soon.

This is another reason why patients have to be considered the centre of IPF research. In their Opinion, Anne-Marie Russell and her colleagues clearly point to the need for putting patients before the condition [9]. Patients, their families and the related not-for-profit organizations have been making and will keep making major positive contributions to the advancement towards a cure. The patients, now becoming true co-investigators, should be at the heart of research to enrich the understanding of what living with IPF is like and to provide a clear direction of travel for the identification of the targets to achieve.

Ultimately, safe and effective drugs reach patients through regulatory agencies. Laura Fregonese and Irmgard Eichler, both working with the European Medicines Agency, describe in their Commentary the failures and the successes in the pathway to approved treatments in IPF [10]. They highlight the potential for improving this process, by learning from mistakes and by expanding the collaborative efforts, with the final aim of having more and better drugs for IPF and ILD patients, including those of paediatric age.

Finally, in the first of the Research Articles in this collection, Simon Walsh and his colleagues explore the complex relationship between pathology and imaging in lung fibrosis [11]. This area of research has been extremely prolific in the last few years and helped to better understand the basic mechanisms for disease maintenance and progression in fibrotic lung diseases. This article provides further support to the concept that traction of airways (as assessed by ‘standard’ chest imaging) is a major determinant of clinical course across a spectrum of fibrotic lung diseases and might be linked to the key pathologic feature of lung fibrosis, that is, fibroblastic foci. These findings will have implications for both prognostications in single patients and the design of future trials of anti-fibrotic treatments.

After the launch of this collection, *BMC Medicine* will remain committed to advancing and spreading knowledge about IPF. In particular, a set of linked articles involving world IPF leaders will address the importance and the limitations of using an evidence-based approach in the management of IPF patients. This issue has enormous potential repercussions, in a global context of increasing attention to the possible areas of clash between guidance and guidelines. Finally, an upcoming Forum article will explore the status of IPF care in a part of the world which is home to the majority of human beings. Four experts, representing the so-called BRIC countries (Brazil, Russia, India and China) will provide, for the first time, an authoritative assessment of the potentials and the pitfalls of managing IPF in these areas of the globe.

We are confident that this article collection will contribute to moving forward towards better care for IPF patients worldwide.

## Abbreviations

ILD: Interstitial lung disease
IPF: Idiopathic pulmonary fibrosis
